# Diode Laser Treatment for Buccal Mucosa Fibrolipoma in an Elderly Patient on Anticoagulants: Case Report and Literature Review

**DOI:** 10.1155/crid/8847648

**Published:** 2025-08-08

**Authors:** Matteo Pellegrini, Martina Ghizzoni, Federica Pulicari, Elisabetta Kuhn, Andrea Scribante, Francesco Spadari

**Affiliations:** ^1^Maxillofacial Surgery and Dental Unit, Fondazione IRCCS Cà Granda Ospedale Maggiore Policlinico, Milan, Italy; ^2^Department of Biomedical, Surgical and Dental Sciences, University of Milan, Milan, Italy; ^3^Pathology Unit, Fondazione IRCCS Cà Granda Ospedale Maggiore Policlinico, Milan, Italy; ^4^Section of Dentistry, Department of Clinical, Surgical, Diagnostic and Pediatric Sciences, University of Pavia, Pavia, Italy

**Keywords:** buccal mucosa, dentistry, diode laser, laser surgery, oral fibrolipoma, oral pathology

## Abstract

**Introduction:** Lipomas are benign neoplasms originating from mesenchymal soft tissue, primarily composed of mature adipocytes and surrounded by a fibrous capsule. While they are relatively common in the head and neck region, oral cavity involvement is rare. Fibrolipoma (FL) is a variant characterized by lobules of adipocytes with dense collagen bands. Its etiology remains uncertain and can occasionally be found in the buccal mucosa. Surgical excision is the gold standard for oral lipomas, and the prognosis is generally favorable. This case report describes the excision of a buccal mucosa FL in a 92-year-old patient undergoing anticoagulant therapy using a diode laser.

**Methods:** A 92-year-old patient with a medical history of benign prostatic hyperplasia and hypertension, under anticoagulant therapy, presented with a painless buccal mucosa swelling. The growth affected both the superficial and submucosal layers. Surgical removal of the FL using a diode laser was performed.

**Results:** The diode laser excision of the FL was carried out successfully, with immediate cauterization, eliminating the need for sutures or hemostatic agents. Histopathological examination confirmed the diagnosis of FL. Postoperative healing was uneventful at both 1- and 3-month follow-ups.

**Conclusions:** Oral FLs are rare benign tumors primarily treated through surgical excision. In this case, the use of a diode laser provided effective hemostasis, minimal scarring, and rapid recovery, making it a suitable option for patients under anticoagulant therapy. While this report presents promising results, further cases with longer follow-up periods are needed to establish the effectiveness and safety of this technique. Laser technology continues to emerge as a valuable tool in oral pathology and surgery, offering minimally invasive alternatives to traditional approaches.

## 1. Introduction

Lipoma is a benign neoplasm that arises from mesenchymal soft tissue. It consists of a central core composed of mature adipocytes that are usually surrounded by a subtle fibrous capsule [1, 2]. While up to 20% of human lipomas are sited in the head and neck region, only 1%–4% of cases affect the oral cavity [3, 4]. Lipoma typically presents as an asymptomatic, well-defined neoplasm with a slow growth pattern. Histologically, fibrolipoma (FL) is considered a lipoma variant enriched with bands of dense collagen that delimit the lobules of mature adipocytes [5, 6]. Its etiopathogenesis remains unknown, with potential causes including endocrine imbalances, congenital factors, fibromatous tumor degeneration, or maturation of lipoblastic embryonic cell nests [7]. In the buccal mucosa, FL can manifest as either a superficial or a submucosal lesion [8]. Rarely, FL can occur on the tongue, lips, palate, retromolar trigone, and floor of the mouth [6, 9, 10]. Oral lipomas seldom relapse after surgical excision, so their prognosis is generally considered favorable [11, 12]. The standard treatment for these neoplasms involves surgical excision or a modern approach using lasers. However, medical treatment involving steroid injections to induce fat atrophy, thus reducing neoplasm dimensions, can also be considered [13]. The aim of this case report is to describe the excision of a buccal mucosa FL in a 92-year-old patient undergoing anticoagulant oral therapy using a diode laser.

## 2. Case Report

### 2.1. Diagnosis and Etiology

A 92-year-old patient was referred to the Maxillo-Facial and Odontostomatology Unit at Fondazione IRCCS Cà Granda Ospedale Maggiore Policlinico in Milan, Italy. The patient had a medical history of benign prostatic hyperplasia and hypertension and was concurrently undergoing treatment with anticoagulant medications (Eliquis, 2.5 mg to be administered twice daily). His current plan includes candesartan, alprazolam, Cardirene, citalopram, Daflon, Avodart, and tamsulosina.

**Figure 1:**
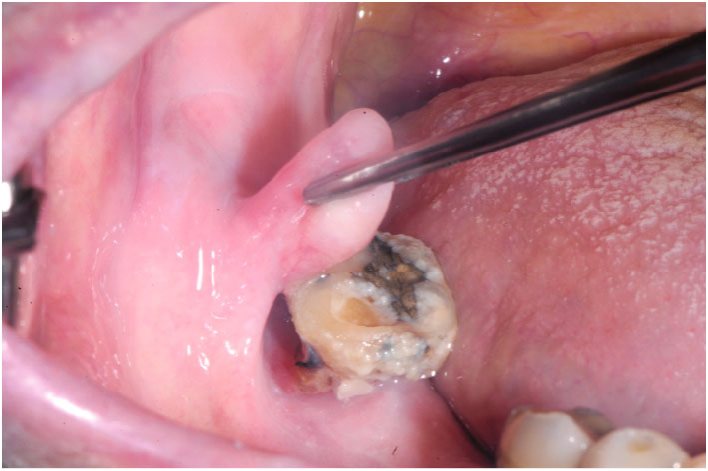
Preoperative appearance of the neoformation.

He reported a painless swelling on the right buccal mucosa, around the second mandibular molar. The swelling became painful when he wore a mobile skeletal prosthesis, hindering its use. The neoformation displayed a pedunculated shape, protruding into both the superficial and submucosal layers of the mucosa, extending upward to the occlusal surface of the mentioned dental element (Figure 1). The neoformation exhibited a soft consistency, well-defined borders, and a normal-colored mucosal covering. After a clinical assessment, a surgical treatment plan, consisting of laser excision, was determined in consultation with the patient. It is essential to consider potential medication interactions when prescribing apixaban, particularly with drugs like Cardirene and citalopram, which may increase the risk of bleeding. Cardirene can enhance the anticoagulant effect of apixaban, while citalopram may impact platelet function. Close monitoring of patients on these combinations is crucial to mitigate bleeding risks. Informed consent was obtained from the patient prior to the excision of the lesion.

### 2.2. Differential Diagnosis

The exophytic lesion presented the possibility of being either reactive or tumoral. A reactive origin was dismissed given the absence of trauma or evident stimulating factors in the patient's medical history, coupled with the vitality of the adjacent teeth. Consequently, the lesion was considered potentially tumoral due to its persistent enlargement without apparent provocation. Given its gradual progression, the tumoral nature was likely benign. Furthermore, its soft consistency raised suspicion of it being a lipoma or fibroma [14].

Because it adheres to surrounding tissues and pseudoinfiltrative features resulting from abundant collagen and connective tissue, this lesion may present challenges in distinguishing it from malignant infiltrating lesions during differential diagnosis [15, 16].

### 2.3. Treatment Objectives

The treatment consists of lesion removal using diode laser surgery with direct wound closure. Laser technology enables direct wound closure by precisely targeting and fusing tissue edges, facilitating rapid and efficient healing [17].

Care was taken to remove the entire neoplasm while avoiding damage to the adjacent anatomical structures, such as facial artery ramifications and lingual nerve.

### 2.4. Treatment Alternatives

The standard method for addressing an oral FL is surgical excision. This procedure involves making an incision in the oral mucosa covering the neoplasm, carefully separating the lesion capsule from the adjacent tissues, and thoroughly eliminating it. Throughout the excision, the surgeon takes measures to ensure effective hemostasis, effectively controlling any bleeding to maintain a clear and controlled surgical environment. Once FL has been successfully removed, the incision is closed using sutures [18]. In this case, a laser excision was performed to avoid bleeding since the patient is anticoagulated.

### 2.5. Treatment Progress

**Figure 2:**
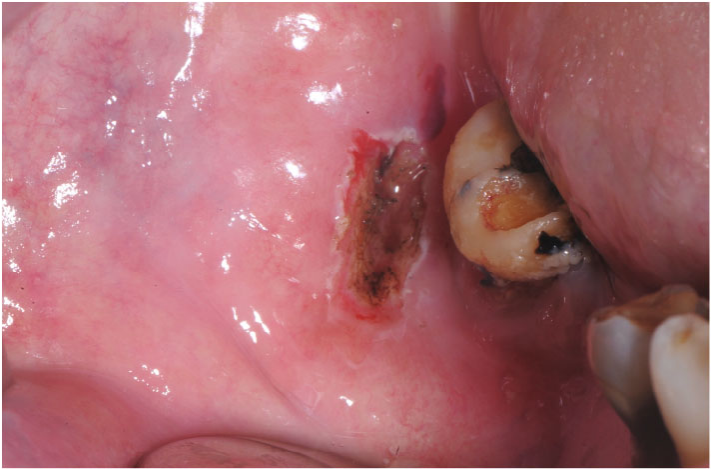
Postlaser biopsy site shows the absence of bleeding (T0).

After administering circumferential anesthesia (mepivacaine + adrenaline 1:100,000, Optocain, Molteni Dental S.r.l., Milan, Italy), the FL was surgically excised using a diode laser, continuous wave, with a tip of 300 nm (Raffaello diode laser, DMT, Lissone, Monza and Brianza, Italy), a wavelength of 645 + 980 nm, and a power of 2.8 W. The cuts were made perpendicular to the insertion point of the pedicle, and the excision extended into healthy tissue to minimize the risk of recurrence (Figure 2, T0 immediate postoperative site), since achieving clear margins ensures the comprehensive eradication of the lesion. With the aid of laser technology, immediate cauterization was performed, eliminating the need for tranexamic acid or sutures. Cauterization settings applied were 445-nm wavelength, 2-J energy, continuous wave, and 1.8-W power.

### 2.6. Treatment Results

**Figure 3:**
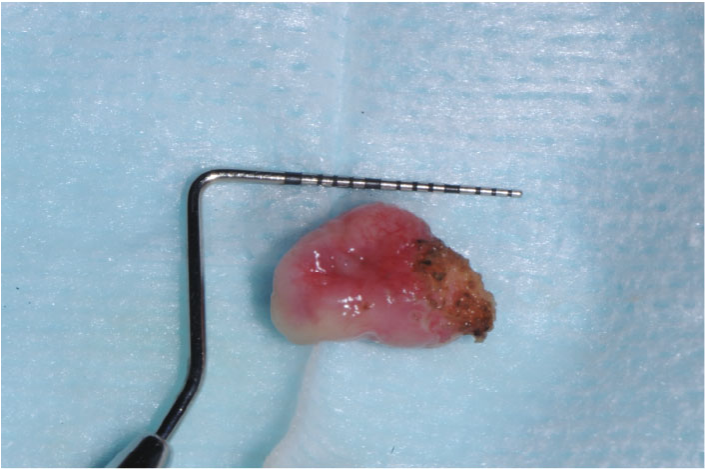
Macroscopical appearance of the surgical specimen.

After FL laser excision (Figure 2), the surgical specimen measuring 1.3 × 0.9 × 0.5 cm (Figure 3) was preserved in 10% buffered formalin and subsequently embedded in paraffin. Hematoxylin and eosin staining was applied. The histological examination confirmed the initial suspicion of FL.

**Figure 4:**
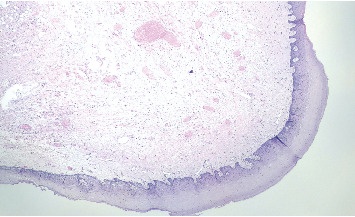
Histologic image of the specimen at 40× magnification.

**Figure 5:**
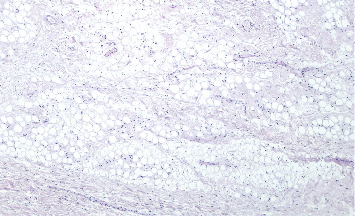
Histologic image of the specimen at 100× magnification.

**Figure 6:**
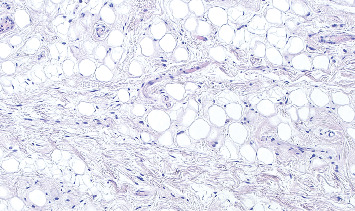
Histologic image of the specimen at 200× magnification.

Representative images of the histopathological features revealed a polypoid mesenchymal lesion covered by squamous epithelium, partially hyperplastic. These images showed ectatic vessels and a fibrous chorion (Figure 4, hematoxylin–eosin stain, 40× magnification). Upon higher magnification, the chorion exhibited abundant adipose tissue (Figure 5, hematoxylin–eosin stain, 100× magnification), characterized by normally appearing adipocytes and scattered inflammatory cells, including rare mastocytes (Figure 6, hematoxylin–eosin stain, 200× magnification).

**Figure 7:**
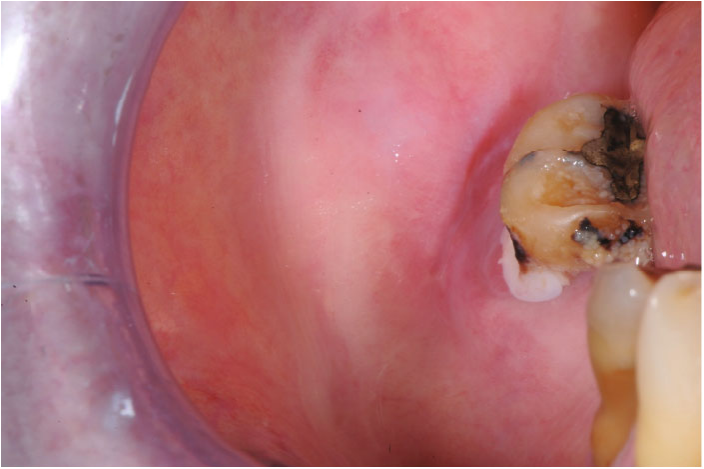
Surgical site 1 month (T1) after laser-assisted surgery.

**Figure 8:**
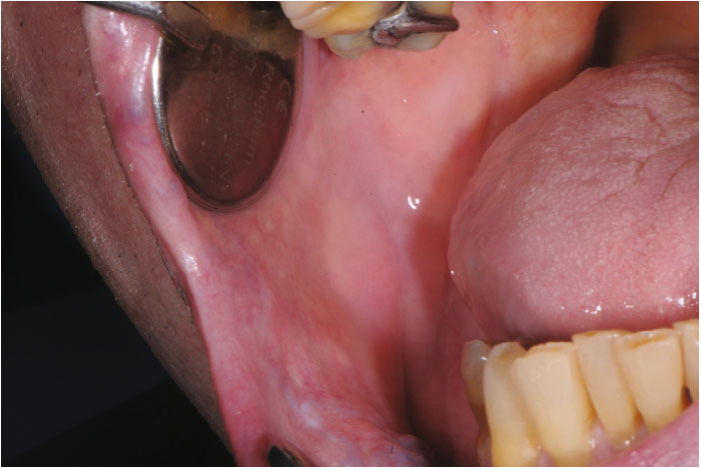
Three months (T2) after laser-assisted surgery.

Postoperative healing occurred without any complications. At T1 (1-month follow-up), the surgical site presented a normally colored mucosa, perfectly healed, without any signs or symptoms of infection (Figure 7). At T2 (3-month follow-up), the surgical site exhibited an esthetically and functionally normal mucosa without any signs or symptoms of infection (Figure 8).

## 3. Discussion

Lipoma is the most common tumor of mesenchymal tissue in adults [19]. In the oral cavity, lipomas are classified into three forms: superficial, diffuse, and encapsulated [20]. These lesions predominantly occur in adults, with some studies suggesting a male predilection [8]. Clinically, oral lipomas present as single, painless nodules, and differential diagnosis is critical, as they may resemble conditions such as fibroma, neurofibroma, or liposarcoma [21]. The buccal mucosa is the most common site, accounting for 30.5%–45.7% of cases due to the presence of the buccal fat pad [16, 22].

Biopsy and histopathological examination are the gold standards for diagnosis [20]. Both lipomas and FLs are treated surgically [23], and although recurrence is rare, careful follow-up is recommended due to the small risk of malignant transformation to liposarcoma [24, 25].

In recent years, diode lasers have become an important tool in oral surgery and pathology. They offer significant advantages over traditional scalpel-based excision, such as intraoperative hemostasis, reduced postoperative bleeding, and faster recovery times [26–31]. This makes laser technology particularly beneficial in patients who are on anticoagulation therapy, as excessive bleeding can be avoided without the need to modify or discontinue anticoagulant medication. Additionally, lasers result in minimal scarring and eliminate the need for sutures, further enhancing patient comfort and reducing recovery time [32–35].

Although only two prior cases of FL excision using diode lasers have been reported in the literature, both involving lesions in the lip and floor of the mouth [35, 36], this case represents the first report of a buccal mucosa FL treated with a diode laser in a patient on anticoagulation therapy [37–43].

Furthermore, although diode laser excision is an established technique for benign oral lesions, the novelty of the present case lies in the unique clinical context. The patient was 92 years old, affected by multiple comorbidities and undergoing chronic anticoagulant therapy (apixaban 2.5 mg BID), alongside medications such as Cardirene and citalopram, which may further increase bleeding risk. In this complex setting, diode laser surgery provided a safe, bloodless, and effective solution without the need to suspend or adjust anticoagulation therapy. To the best of our knowledge, the literature lacks detailed case reports of buccal FLs managed with diode lasers in similarly fragile and polymedicated geriatric patients.

The diode laser was selected for this procedure due to its ability to precisely excise the lesion while minimizing intraoperative bleeding, making it the optimal choice given the patient's clinical condition. Laser therapy offers several significant advantages over traditional surgical approaches for excising lesions. The precision of laser technology allows for targeted tissue removal while minimizing damage to surrounding healthy tissues. Additionally, laser therapy promotes intraoperative hemostasis, significantly reducing bleeding and the need for sutures, which results in quicker recovery times and less postoperative discomfort for patients. The use of lasers also diminishes scarring, enhancing esthetic outcomes, particularly in sensitive areas such as the oral cavity. This underscores the utility of laser technology in managing complex cases, offering a less invasive and more efficient alternative to conventional surgical methods.

Table 1 shows a summary of the literature focusing on cases of oral FLs found in the buccal mucosa.

However, this case report has some limitations. It describes only a single case treated with this approach and has a relatively short follow-up period. More cases with long-term follow-up are necessary to establish the success of this technique. Additionally, it is important to note that laser equipment entails a significant investment and requires a learning curve for the operator to achieve optimal surgical results.

## 4. Conclusion

The use of a diode laser for excising benign lesions in the oral mucosa represents a modern approach when contrasted with traditional blade surgery. In our patient's case, this method facilitated a minimally invasive removal of the FL, effectively preventing intraoperative bleeding and minimizing both scarring and recovery time.

## Figures and Tables

**Table 1:** Review of case reports of fibrolipoma of the buccal mucosa.

**References (authors and year of publication)**	**Age (years or mean age)**	**Gender**	**Location**	**Histopathological diagnosis**
Manjunatha et al., 2010 [1]	55–75 years, mean age: 65 years	2 M	Right buccal mucosa	Fibrolipoma
Studart-Soares et al., 2010 [3]	46.6 mean age	2 F, 1 M	Buccal mucosa	Fibrolipoma
Epivatianos et al., 2000 [7]	40 years	M	Buccal mucosa	Fibrolipoma
Chhetri et al., 2022 [21]	68 years	Male	Right buccal mucosa	Fibrolipoma
Manor et al., 2011 [22]	59.7 mean age	N.R.	Buccal mucosa	Fibrolipoma
Fregnani et al., 2003 [16]	N.R.	N.R.	Buccal mucosa	Fibrolipoma
Iwase et al., 2016 [23]	71 years	M	Left buccal mucosa	Fibrolipoma
Khubchandani et al., 2012 [37]	10 years	Female	Left buccal mucosa	Fibrolipoma
Wu et al., 2020 [38]	43 years	F	Left buccal mucosa	Fibrolipoma
Perez-Sayáns et al., 2019 [39]	56.4 mean age	N.R.	Buccal mucosa	Fibrolipoma
Juliasse et al., 2010 [40]	56.7 mean age	N.R.	Buccal mucosa	Lipoma (41.5%), fibrolipoma (34.1%), spindle cell lipoma (9.8%), sialolipoma (9.8%), osteolipoma (2.4%), and chondrolipoma (2.4%)
Janas et al., 2005 [41]	60 years	M	Buccal mucosa	Fibrolipoma

## Data Availability

The authors confirm that the data supporting the findings of this study are available within the article.
